# Editorial: The Incredible Challenge of Digitizing the Human Brain

**DOI:** 10.3389/fpsyg.2022.808275

**Published:** 2022-02-21

**Authors:** Luciano Di Mele, Carmen Moret-Tatay, Mike Murphy, Céline Borg, Raúl Espert-Tortajada, Camila R. De Oliveira

**Affiliations:** ^1^Facoltá di Psicologia, International Telematic University UNINETTUNO, Rome, Italy; ^2^Departamento de Neuropsicobiología, Metodología y Psicología Social, Universidad Católica de Valencia San Vicente Mártir, Valencia, Spain; ^3^Dipartimento di Neuroscienze, Salute Mentale e Organi di Senso (NESMOS), La Sapienza University of Rome, Rome, Italy; ^4^School of Applied Psychology, University College Cork, Cork, Ireland; ^5^CMRR Neuropsychologie Chu de St Etienne, Equipe Vision et Cognition Laboratoire de Psychologie et Neurocognition CNRS 5105 Université UGA, Université Catholique de Lyon, Lyon, France; ^6^Departament de Psicobiologia, Universitat de Valéncia, Valencia, Spain; ^7^Mestrado em Psicologia, IMED, Passo Fundo, Brazil

**Keywords:** social media, media education, multitasking, human cognition, informational nature, digitalization

The world we live in is drastically different from previous decades in terms of digitalization expansion. Logically, one might expect that these changes modify the way we behave, our habits, the way we do tasks, communicate and access information (Moret-Tatay et al., 2018; Wang et al., 2021). And, therefore, the functioning of the brain and even its anatomy. If so, it seems imperative to examine how it affects our cognition. Furthermore, based on the assumption that the brain is plastic and adaptable, some alterations and changes are expected to optimize resources or even compensations by improving other skills (Oliveira et al., 2018; Bubbico et al., 2020; Della Gatta et al., 2021).

It should be noted that these developments take place in the context of a rapidly aging society. Therefore, age-related differences in this technology adoption process are expected, as is the case in previous literature (Charness et al., 2018). Due to the historical moment we live in, we face a once-in-a-lifetime opportunity to address these issues by including theoretical, methodological, and empirical contributions. These might shed light on the following question underlying the current Research Topic: (i) models for human cognition and challenges for the human brain in communication with the digital society; (ii) how media multitasking affects our mental processes.

First, from a theoretical perspective, the first contribution (Byrne) describes a pendulum-like approach to neuron interactions: this involves the rapid firing and restarting of the process and the clusters of neurons in circuits. A digital analogy is proposed through electroencephalogram (EEG) techniques to show frequency changes that are characteristic of different cognitive processes (Golnar-Nik et al., 2019). In this way, each pendulum would represent a process in terms of length, weight, and a damping factor, previously described in terms of quantum search (Chen and Brylinski, 2002). Thus, this approach highlights the field of quantum theory to model cognitive phenomena underlying the information processing by the human brain and its cognitive components such as language, perception, or memory, among others (Jedlicka, 2017). Behavior studies suggest environmental changes, novel or ambiguous situations, which might support an analog new paradigm designed to provide better insights into our subconscious decision-making.

From a behavioral perspective, and according to Ruiz-Ruano García and Puga, our brain is considered a cognitive system with limited processing capacity, and therefore overload can occur. More social aspects are framed as the repercussions for mental health, resulting from increased demand for virtual communication. This may involve other fields such as education and their competencies to search, filter and use high quality online documentation, also described in previous literature (Mangen, 2010, 2016). It is clear that this issue is complex and involves many agencies such as communication channels and the education system. Notably, the latter is called on to provide new generations with adequate Information Literacy. On the other hand, this research emphasizes the need to examine the differences in performance between digital and traditional media. In this way, the study carried out by Bernabe-Valero et al. sought to examine differences in response between face-to-face and virtual users, supporting previous literature. According to the authors, similar scores are found in both environments supporting the ecological validity of instruments developed in the present such as the G20.

Another view to examine benefits and side effects has been addressed by Popławska et al. This hypothesis and theory article seeks to analyze the conditions of multitasking. According to the authors, when assessing the effectiveness of media multitasking, it is necessary to specify the reference objectives, consequences, and inherent performance. This approach makes it possible to examine multitasking as a strategic behavior undertaken as well as a failure of self-regulation. Digital media has redesigned the way the mind creates innovative social connections. Social communities have multiplied thanks to the web, and thus novel collective resources have contributed to solving problems, sharing skills and creating emerging communities.

Lastly, Beuckels et al. address the role of multitasking in today's society and underlying issues in multitasking. Thus, this piece of research provides a state-of-the-art investigation into multitasking through a systematic review considering the significant increase in papers on this subject in recent years. The primary purpose is to clarify the difference between multitasking and media multitasking and identify the main research trends. A high burden on cognitive resources is described, as suggested by previous literature on this Research Topic, and a review of state of the art is proposed, using a quantitative method, employing a bibliometric and thematic content analysis to identify five major research themes and trends in the general field of media multitasking. The authors identify methodological problems (e.g., differences between survey techniques and cortical measurement of human activity), group differences (highlighting the role of adolescents in this field), theories (e.g., shallowing hypothesis) and types of media.

Digitizing the human brain is still an open challenge, but two aspects are to consider. Firstly, the trap of technological determinism must be avoided (Moret-Tatay and Murphy, 2019). The media identify themselves as the only instigators behind the current historical transformation. Secondly, interdisciplinarity is necessary; the study of mind and digital technologies needs to converge several sciences such as psychology, sociology, neuroscience, education, and philosophy. From current results, it can be concluded that new challenges in the digitization of the human brain will involve changes in theoretical perspective, including the differences between digital and analog approaches, studies on the saturation of cognitive processes and multitasking, breakdowns by groups and media types, as well as the study of ecological validity. In sum, the debate is often framed in dualistic terms, whether the mind or the technology is superior. When combined with the digital realm, the mind forms a continuous dimension; it no longer makes sense to differentiate online or offline. Currently, this unification of personal and social identities, exaggerated by digital media, constitutes a unique form in its informational nature (Floridi, 2014). This unified identity produces emergent behaviors in numerous fields of human life, with outcomes that must be constantly studied and addressed. However, scientific research often focuses on the risks and opportunities of digitalization. Instead, it would be appropriate to broaden the scope of this research to understand this phenomenon in its broader context for all ages (Moret-Tatay et al., 2019). The manuscript selection described in this Research Topic covered many highly complex topics related to this emerging phenomenon.

## Author Contributions

All authors listed have made a substantial, direct, and intellectual contribution to the work and approved it for publication.

## Conflict of Interest

The authors declare that the research was conducted in the absence of any commercial or financial relationships that could be construed as a potential conflict of interest.

## Publisher's Note

All claims expressed in this article are solely those of the authors and do not necessarily represent those of their affiliated organizations, or those of the publisher, the editors and the reviewers. Any product that may be evaluated in this article, or claim that may be made by its manufacturer, is not guaranteed or endorsed by the publisher.
